# Using Effort to Measure Reward Value of Faces in Children with Autism

**DOI:** 10.1371/journal.pone.0079493

**Published:** 2013-11-13

**Authors:** Louise Ewing, Elizabeth Pellicano, Gillian Rhodes

**Affiliations:** 1 Australian Research Council Centre of Excellence in Cognition and its Disorders, School of Psychology, University of Western Australia, Perth, Australia; 2 Centre for Research in Autism and Education (CRAE), Institute of Education, University of London, London, United Kingdom; Ecole Normale Supérieure, France

## Abstract

According to one influential account, face processing atypicalities in autism reflect reduced reward value of faces, which results in limited attention to faces during development and a consequent failure to acquire face expertise. Surprisingly, however, there is a paucity of work directly investigating the reward value of faces for individuals with autism and the evidence for diminished face rewards in this population remains equivocal. In the current study, we measured how hard children with autism would work to view faces, using an effortful key-press sequence, and whether they were sensitive to the differential reward value of attractive and unattractive faces. Contrary to expectations, cognitively able children with autism did not differ from typically developing children of similar age and ability in their willingness to work to view faces. Moreover, the effort expended was strongly positively correlated with facial attractiveness ratings in *both* groups of children. There was also no evidence of atypical reward values for other, less social categories (cars and inverted faces) in the children with autism. These results speak against the possibility that face recognition difficulties in autism are explained by atypical reward value of faces.

## Introduction

Reduced or atypical looking at faces is often reported in children with autism [1]–[3], as are a range of face processing difficulties (see [4], [5]). Yet after thirty years of research in this area, the mechanisms underlying these differences remain unclear. One influential proposal attributes these difficulties to atypical social motivation and reduced reward value of social stimuli for individuals with autism [6]–[10]. Decreased reward value of social stimuli, such as faces, early in development is proposed to reduce attention to these stimuli, diminish motivation to engage in reciprocal social interactions and limit the acquisition of processing expertise.

Interestingly, despite increasing interest in reward processing generally in autism (including a recent thematic series in the *Journal of Neurodevelopmental Disorders*, 2012), few studies have *directly* investigated the reward value of faces per se. Moreover, these few existing studies have yielded mixed results. With regards to neuroimaging findings, some researchers have reported that, relative to typical children and young people, individuals with autism show selectively atypical neural activity in regions associated with rewards (e.g. ventral striatum, left dorsal striatum) when receiving social rewards, such as having a smiling face on-screen, but not monetary rewards [11] and when receiving socially valenced feedback (smiling vs frowning faces) during a learning task [12]. There is also some preliminary support for selectively increased pupil dilation responses to faces (smiling with direct gaze) in typical children, but not in children with autism [13], which may be linked with reward outcomes [14]. These findings are all consistent with diminished face rewards in autism.

Yet, other findings challenge the notion of selectively atypical social rewards in autism. For example, Kohls and colleagues presented electrophysiological [15] and imaging evidence [16] of broadly diminished neural reward responses to both monetary *and* social incentives in individuals with autism, relative to typical individuals. In another example, the findings are precisely the opposite of predictions from reward accounts. Dichter et al. [17] reported comparable social reward-circuitry activation in adults with autism and typical participants in response to viewing faces, but selectively diminished rewards associated with *monetary* incentives in the autism group.

Behavioral evidence seems to paint a similar picture of intact reward value of faces in autism. Attractive faces have higher reward value than unattractive faces [18], [19] and typical adults will work harder to view them [20]. Recent reports of typical perceptions of attractiveness in children and adults with autism suggest that sensitivity to differences in reward value associated with differences in facial attractiveness may be unaffected in individuals with the condition [21], [22], but see [23]. Yet, the possibility that standards of beauty can be learned [24] as well as the extent to which these self-report ratings require interpretation of subjective feeling states may compromise the validity of attractiveness as an index of stimulus reward value in individuals with autism.

The limited, inconclusive research into face rewards in autism to date clearly warrants further investigation of this issue. Here, we draw upon the principles of behavioral economics to measure stimulus reward value. A defining characteristic of a rewarding or reinforcing stimulus is that it motivates behavior [25]. We therefore adapted a key pressing task from behavioral economics [20] to create a developmentally appropriate task to index the reward value of stimuli by measuring the effort children were willing to expend in order to view them. In so doing, we aimed to test directly the reduced reward hypothesis [7], [8] – that the reward value of faces is diminished during development in individuals with autism – by investigating whether children with autism are less motivated to view faces than typically developing children. We also sought to determine whether children with autism would be relatively less sensitive to variations in the reward value of these social stimuli, by examining our participants’ willingness to expend differing degrees of effort for faces varying in attractiveness.

We further examined children’s responses to cars and inverted faces. The inclusion of inverted faces, which have similar low-level visual features to faces but are less socially relevant, allowed us to investigate whether any reduction in reward value of (upright) faces might reflect their social significance. Cars, which have been used in other research investigating the selectivity of face processing atypicalities in autism (e.g., [26]), served as a non-social, second perceptually homogeneous category of comparison stimuli. According to the reduced reward hypothesis, we predicted that children with autism should ‘work’ less and therefore make fewer key presses than typical children to view faces, but not cars or inverted faces.

## Methods

### Ethics Statement

The study was approved by the Human Research Ethics Committee at the University of Western Australia and all parents provided written consent prior to their child’s participation in the project. All children also gave verbal assent before taking part and some older children and adolescents also provided written consent.

### Participants

Nineteen cognitively able autistic children (17 boys) aged 8 years 0 months to 15 years 0 months, were recruited from local schools, community groups and the West Australian Register for Autism Spectrum Disorders (see Table 1). These children were independently diagnosed with Autistic Disorder (n = 15), Asperger’s Syndrome (n = 3) or Pervasive Developmental Disorder Not Otherwise Specified (n = 1) by a multidisciplinary clinical team following DSM-IV criteria [27]. Parents completed the Social Communication Questionnaire (SCQ [28]), a retrospective questionnaire measure of their child’s autism symptomatology (n = 15). All parents rated their child at or above the cut-off for clinically-significant levels of autistic symptomatology (score of 15). Participants also completed either Module 3 or 4 of the Autism Diagnostic Observation Schedule – Generic (ADOS-G [29]). All children scored above the autism spectrum algorithm cut-offs, which indicated that their levels of current autistic symptomatology were sufficient to meet ADOS-G criteria for the condition.

**Table 1:** Chronological Age, Cognitive Ability, SCQ, ADOS-G and CFMT-C scores for children with autism and typically developing children.

	Group				
	Autism (n = 19)		Typical (n = 19)		
Measure	Mean (SD)	Range	Mean (SD)	Range	
**Age** *(months)*	136.7 (26.9)	96–180	136.3 (32.2)	91–179	*t*(36) = 0.04, *p* = .96, *d* = .01
**Non-verbal IQ^a^**	98.2 (14.3)	73–129	96.8 (7.6)	82–106	*t*(36) = 0.36, *p* = .71, *d* = .12
**Verbal IQ^a^**	100.0 (12.2)	77–124	101.3 (8.8)	83–114	*t*(36) = 0.34, *p* = .72, *d* = .11
**SCQ^b,c^**	25.2 (5.5)	16–36	3.3 (3.3)	0–10	*t*(31) = 14.26, *p*<.001, *d* = 5.12
**ADOS-G^b^**	10.3 (2.5)	7–17			
**CFMT-C^d^**	43.0 (7.0)	28–52	47.5 (6.0)	31–56	*t*(36) = 2.12, *p*<.05, *d* = .70

Notes. **^a^**Non-verbal and Verbal IQ were each measured with two subtests of the WISC-IV (Wechsler, 2003); Non-verbal IQ = Matrix Reasoning and Picture Completion, Verbal IQ = Similarities and Vocabulary. ^b^Higher scores on both the parent-report Social Communication Questionnaire (SCQ); Rutter et al., 2003) and the ADOS-G (Autism Diagnostic Observation Schedule – Generic, Lord et al., 2000) indicate a greater degree of autistic symptomatology. Score reported = Communication+Social Interaction algorithm total (cutoffs: autism = 10, autism spectrum = 7). ^c^n = 15 for the autism sample, n = 18 for the typical sample. ^d^Accuracy (total correct) scores on the Cambridge Face Memory Test – for Children (maximum = 60).

Nineteen typically developing children and adolescents (17 boys) recruited from local schools were well matched to the autistic sample on chronological age, non-verbal IQ and verbal IQ (*p*s>.71; Table 1). No typical participant had a history of psychiatric/neurological disorder as reported by parents, or displayed clinically-significant levels of autistic symptomatology as indexed by scores below the cut-off on the SCQ. Face recognition was impaired in the children with autism relative to this typical group, as indicated by lower scores on the Cambridge Face Memory Test for Children (CFMT [30]) (see Table 1). This measure is adapted from the standardized adult face memory measure [31] with a reduced test set (5 rather than 6 to-be-remembered items) and fewer foils (participants make 2-alternative rather than 3-alternative forced choice decisions) to be more appropriate for children.

### Stimuli

Color images of 40 female faces (direct gaze, neutral expression) and 20 cars (angled 45 degrees right) were selected from the Internet to span a large range of attractiveness (confirmed by participant ratings below). These images were standardized for size (average visual angle approximately 6.9°×5.7° for faces; 8.0°×3.4° for cars) for use in a child-friendly reward task (The Viewing Game) adapted from [20]. The backgrounds of each car image were colored black and black oval masks were placed around the external contour of each face to cover most of the hair. Alternate versions of the face images, rotated 180 degrees, were created for use as inverted faces. The assignment of faces to the upright and inverted orientation conditions within the task was counterbalanced across participants within each group.

### Procedure

The Viewing Game was administered on a 15-inch MacBook Pro laptop computer as part of a larger battery of behavioral tasks, cognitive ability tests, and the ADOS-G. Testing was conducted in a quiet room at home or at the University, over two or three 90–120 minute activity sessions.

Children were told that during the Viewing Game they would be presented with lots of different pictures. They could control the number of times they viewed each one, by completing a key pressing sequence on the keyboard (“z” then “p” with the same finger). If they wanted to see an image once, twice, or any number of times, it was their choice to do so. It was made clear that they were free to press the keys as many or as few times as they wished and, importantly, that the amount of key pressing they performed on a given trial would not affect the overall length of the game.

On each trial, a space-bar press initiated the presentation of an image (face, car or inverted face) for 800 ms. This image was then replaced by a blank screen. If the child wanted to briefly see the image again, they were instructed to complete the effortful “z” “p” key pressing sequence, which would bring the image up on screen again for an additional 500 ms. This key pressing sequence could be repeated any number of times within the duration of the trial (5 seconds), when a blank screen and a chime cued participants to press the space-bar to reveal the next stimulus. If no keys were pressed during a trial, the screen remained blank until the end of the 5 second trial duration.

Participants were initially familiarized with the task requirements with four extended demonstration trials with dog images (10 seconds each). During these trials, children were encouraged to experiment with pressing and not-pressing the response keys. They then completed 6 practice trials (2 faces, 2 cars, 2 inverted faces) that matched the structure of the test trials (5 second trials). The main task consisted of three 20-trial blocks. Here, trials with face, car and inverted face images were intermixed and presented in one of two randomized orders, counterbalanced between participants. Allocation of face stimuli to the upright and inverted orientation conditions was also counterbalanced between participants.

After completing the Viewing Game, children were shown the images again in a random order, each for an unlimited duration. They were instructed to rate them for how “attractive or good looking” they were, using a 5-point Likert scale consisting of five numbered cups of increasing size (1 = “really unattractive, bad looking, or ugly”, 5 = “really attractive, good looking, or beautiful”; [32]).

## Results

**Figure 1:**
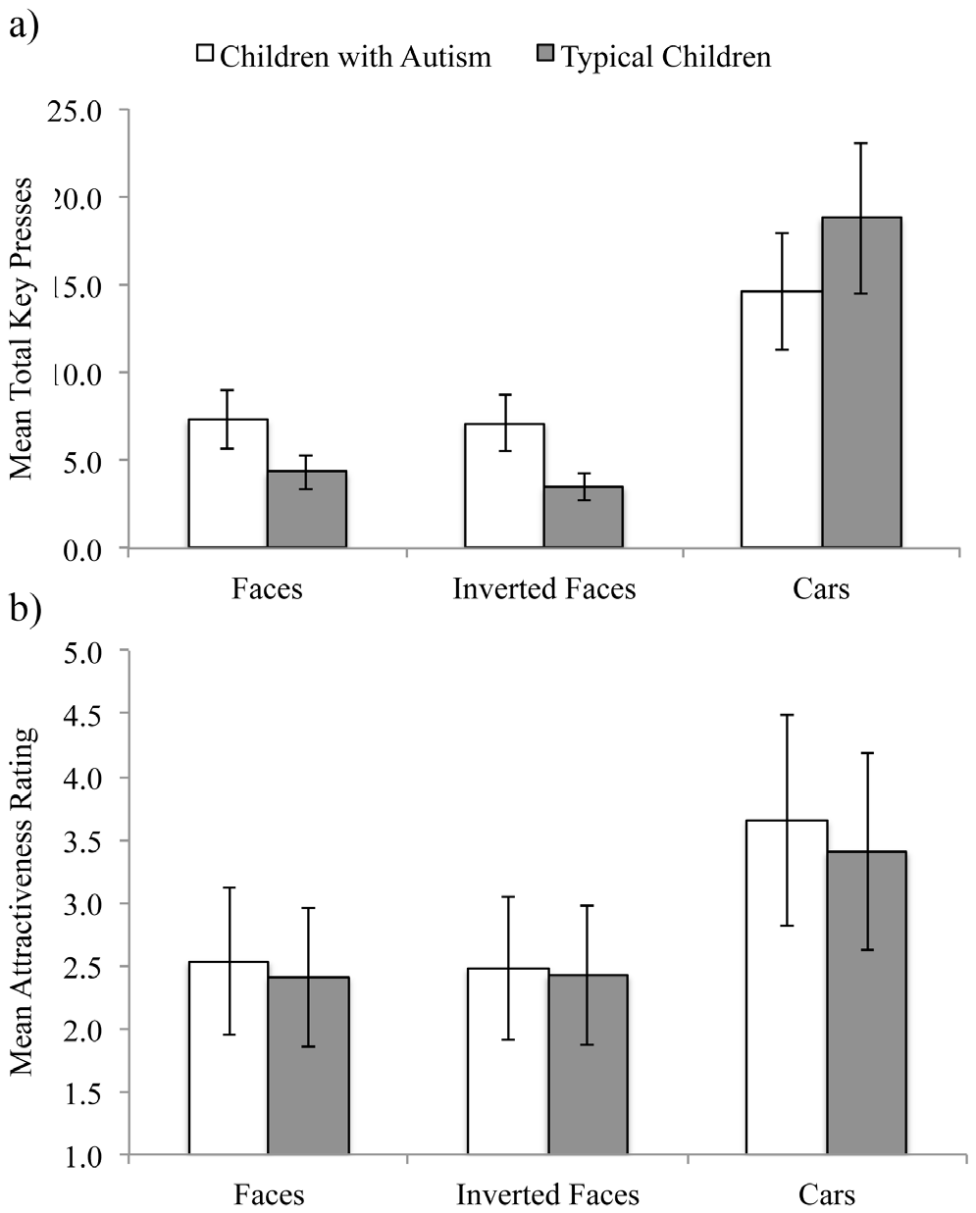
Key presses and attractiveness ratings. Mean (+SEM) total key presses (a) and attractiveness ratings (b) for each stimulus category and group are shown. As expected, these values appear ‘moderate’ (e.g., in the mid-range of the attractiveness rating scale) because they reflect participants’ averaged responses to images spanning a range of attractiveness levels.

Our index of stimulus reward value was the total number of key presses (i.e. “z” “p” key pressing sequences completed) for each stimulus category. With this dependent variable, there was no evidence that the reward value of faces was significantly diminished for children with autism, *t*(36) = 1.28, *p = *0.20, who actually showed numerically slightly more key presses to faces than typically developing children (see Figure 1A). A two-way ANOVA with group (autism, typical) as a between-participants factor and stimulus type (faces, inverted faces, cars) as a repeated-measures factor on children’s total number of key presses confirmed that there was no main effect of group, *F*(1, 36) = 0.15, *p* = .69, *partial η^2^* = .01.

There was a significant effect of stimulus type, *F*(2, 72) = 75.01, *p*<.01, *partial η^2^* = .67, with significantly more key presses to cars (M = 16.6, SD = 8.3) than faces (M = 5.8, SD = 7.1), *t*(37) = 7.68, *p*<.01, or inverted faces (M = 5.3, SD = 6.6), *t*(37) = 8.32, *p*<.01, which did not differ from each other, *t*(37) = 1.55, *p = *.12. We speculate that these differences may reflect the interests of the young, predominantly male participants in both groups and/or our stimulus selection. There was also a significant interaction of stimulus type with participant group, *F*(2, 72) = 8.52, *p*<.01, *partial η^2^* = .19. Importantly, however, this result did not reflect selectively reduced key pressing for faces by the children with autism. There were no significant group differences in key pressing for any stimulus category, all *t*s<1.75; all *p*s>.09. Instead, this interaction seemed to reflect (non-significantly) increased key pressing in the autism group, relative to the typical group, for faces and inverted faces but not cars (see Figure 1).

Key presses did not correlate significantly with age, verbal ability (summed raw verbal subtest scores), non-verbal ability (summed raw performance subtest scores) or face memory (CFMT total score) in either participant group for any of the stimulus categories, all τs<.22, *p*s>.10.

Next, we examined children’s attractiveness ratings, which showed a similar pattern to the key pressing results (see Figure 1B). There was no main effect of participant group, *F*(36) = .79, p = .37, *partial η^2^* = .02. Children with autism did not rate faces as less attractive than typically developing children, *t*(36) = 0.58, *p* = 0.56. There was a significant effect of stimulus type, *F*(2, 72) = 71.66, *p*<.01, *partial η^2^* = .66, with cars (M = 3.5, SD = 0.1) rated more attractive than faces (M = 2.4, SD = 0.1) and inverted faces (M = 2.5, SD = 0.1) both *p*s<.001 (see Figure 1B), which did not differ from each other, *t*(37) = .32, *p* = .74. There was no significant interaction of stimulus type with participant group, *F*(2, 72) = .45, p = .63, *partial η^2^* = .01.

We then assessed whether key pressing behavior in each participant group was sensitive to differences in reward value associated with variation in attractiveness within each type of stimulus. We therefore correlated the mean number of key presses with mean attractiveness ratings (averaged across participants) across the individual items in the face, car and inverted face sets. Attractiveness ratings in the two participant groups were highly internally consistent (all Cronbach’s α>.77) and were strongly correlated for faces, τ(38) = .76, *p*<.001, inverted faces τ(38) = .67, *p*<.001, and cars τ(18) = .85, *p*<.001. As expected, in the typical group, number of key presses correlated strongly with rated attractiveness for faces τ(38) = .44, *p*<.001, cars τ(18) = .86, *p*<.001, and inverted faces τ(38) = .58, *p*<.001, confirming that children, like adults [20], will work harder to view more attractive images and validating this key pressing task as an index of stimulus reward value for children. Most strikingly, these correlations were also strong for children with autism for faces τ(38) = .58, *p*<.001, cars τ(18) = .65, *p*<.001, and inverted faces τ(38) = .59, *p*<.001, indicating sensitivity to variations in reward value associated with attractiveness for all three categories.

### Relationship with Symptom Measures

We also investigated whether our behavioral measure of face rewards was associated with autism symptomatology in our sample. This analysis revealed no significant correlation between total key presses for faces and children’s current (ADOS-G), τ(17) = −.07, *p* = .67, or lifetime symptoms (SCQ) τ(13) = .37, *p* = .07 (Bonferonni correction for multiple comparisons, α = .025). Similarly, when each child’s key pressing for faces was relativized to their key pressing overall (total key presses for faces/total key presses for all three categories), there remained no correlation with either of these symptom measures, all *τ*s<.20, *p*s>.35.

## Discussion

Contrary to the reduced reward hypothesis [7], [8], [10], we found no evidence of atypical reward value for faces in children with autism. Our measure revealed that a group of cognitively able autistic children with significantly impaired face memory (indexed via scores on the Cambridge Face Memory Test for Children) were no less motivated than typically developing children to view faces and were highly sensitive to variation in the reward value of faces associated with differences in attractiveness. These behavioral results provide no support for the view that currently reduced rewards contribute to the face processing difficulties observed in children with autism.

These findings cannot be dismissed due to concerns regarding statistical power. The non-significant effects of participant group were associated with very small effect sizes. Moreover, children with autism made numerically slightly *more* key presses than typically children (a non-significant difference), signaling that the direction of any group difference was opposite to that predicted by the reduced reward hypothesis. This profile of slightly elevated responding, relative to typical children, also allays concerns that atypical motor co-ordination in the children with autism (not assessed here, but widely reported, see [33]) might have undermined successful execution of the required motor response.

As in [20], we interpret our behavioral measure as an indication of stimulus reward value. Yet there are other possible interpretations of the behavioral (key pressing) response, including preferences for (1) novelty or (2) familiarity. We believe, however, that the observed data is inconsistent with these two possiblities. A novelty preference is unlikely because the most novel stimulus category, inverted faces, failed to elicit more key presses than faces or cars for either participant group. A preference for familiarity, which could trigger repeated key presses for stimuli irrespective of reward value, is also at odds with the close alignment of behavioral responses and attractiveness ratings in each group. On balance, we suggest that the key pressing measure reflects a valid behavioral index of stimulus reward value.

The absence of behavioral atypicalities in the current sample does not preclude the possibility of atypicalities in their underlying neural circuitry associated with face rewards. Indeed, several studies report typical behavioral responses alongside atypical neural activation to face rewards in individuals with autism (e.g., [11], [15], [16]). Nevertheless, our results are consistent with at least one recent report of comparable neural reward-circuitry activity associated with viewing static faces in adults with and without autism, along with intact behavioral reward responses [17]. These data and our key pressing results constitute converging evidence that face images may be no less rewarding for individuals with autism, than for typical individuals. Such findings prompt consideration of alternative accounts of the origins of face processing difficulties in children with autism, such as other social processing deficit theories that do not assume diminished face rewards (e.g., 'fast-track modulator model', [34]) and accounts that propose a non-social origin of face processing difficulties, such as a detail-focused processing style [35].

It is possible, of course, that the current sample might have shown atypicalities in their face rewards earlier in development. The social motivation account emphasizes the potential impact of reduced attention to faces during critical periods developmentally preceding the age range studied [6] and emerging neuroimaging evidence supports the possibility of changes in responsiveness to social stimuli, such as faces, following intensive behavioral intervention (e.g., [36]). Future studies should investigate reward processing in autism with a prospective longitudinal design to allow assessment of any changes in the reward value of faces during development, as well as their impact on emerging cognition and behavior.

It was interesting that children across both groups expended more effort to view cars than faces. This profile of key pressing suggested that all participants found viewing car images more rewarding than viewing faces, which was confirmed directly by their attractiveness ratings. At first glance, this result may seem counterintuitive, given the putative ‘special’ status of faces as a highly rewarding, socially informative stimulus category [18], [20], [37]. We speculate, however, that our finding may simply reflect the preponderance of boys in our sample who typically prefer to play with cars than dolls (see also [38] for detailed discussion of sex differences in car interest and expertise). It could also reflect a stimulus selection effect. We made no attempt to equate the attractiveness of stimuli *between* categories because our primary interest was in whether there were any significant group differences in the reward values of stimuli *within* categories.

In line with previous evidence of intact attractiveness perception in autism [21], [22], we observed strong, significant correlations between the (highly consistent) attractiveness ratings made by the children with and without autism for all three stimulus categories. This result suggests successful translation of subjective feeling states to self-report judgments in these children with autism. We note here that intact attractiveness perception is not inconsistent with the social motivation theory of autism, when considered within an evolutionary framework. Chevallier et al. [6] recently proposed that some interpersonal dispositions, such as sexual drive and attachment, might be spared in individuals with the condition because they result from pressures distinct from those driving social affiliation. Somewhat unexpectedly, participants’ mean attractiveness ratings of upright and inverted faces did not differ significantly in either group. This pattern of results contrasts with previous evidence that inversion can disrupt attractiveness judgments (e.g., [39], [40]) but is consistent with our stimuli having been carefully sourced from the Internet to include unambiguously attractive and unattractive exemplars (e.g. models and individuals used in campaigns warning of the effects of long-term drug use). Some characteristics important for attractiveness judgments, such as symmetry, may have been harder to detect when these images were presented inverted (see [41]). Nevertheless, it appears that their aesthetic appeal from other cues, such as skin color and texture, was sufficiently ‘obvious’ to be judged equally in both orientations.

Given that much of the evidence for atypical face processing in autism comes from computerized tasks, it is important to ask whether the reward values of faces are reduced under such conditions. We found no evidence of group differences in reward values for face, car, or inverted face images. It remains to be seen, however, whether face rewards remain typical for autistic children for dynamic stimuli, faces showing emotional expressions, or in more natural settings, such as live social interactions, which may be more arousing and possibly even aversive [42]. In our task, participants had the option on each trial of viewing a test stimulus or a blank screen. It is possible that a different behavioral profile might emerge when alternative viewing objects are available. Moreover, faces represent just one, albeit critical, example of a social stimulus. Future research should investigate the exent to which individuals with autism will work to access other potentially social rewarding stimuli, such as voices and bodies. It will also be important to investigate how the current findings generalize beyond our so-called ‘high-functioning’ clinical group because it seems plausible that social motivation difficulties might present differently in individuals with more severe social and learning difficulties.

In summary, contrary to the reduced reward hypothesis, we found no behavioral evidence of atypical reward values for faces, or indeed other stimulus categories, in autism. Cognitively able autistic children with poor face memory seemed “tuned in” to variations in the reward value of more and less attractive faces, inverted faces and cars, and worked as hard as typical children for the opportunity to view these stimuli.
